# Decrease of 14–3-3 proteins by glutamate exposure in the cerebral cortex of newborn rats

**DOI:** 10.1186/s42826-020-00041-5

**Published:** 2020-04-03

**Authors:** Ju-Bin Kang, Seung-Yun Lee, Dong-Ju Park, Phil-Ok Koh

**Affiliations:** Department of Anatomy, College of Veterinary Medicine, Research Institute of Life Science, Gyeongsang National University, 501 Jinju-daero, Jinju, 52828 South Korea

**Keywords:** 14–3-3 proteins, Cerebral cortex, Glutamate, Neonate

## Abstract

Glutamate is a representative excitatory neurotransmitter. However, excessive glutamate exposure causes neuronal cell damage by generating neuronal excitotoxicity. Excitotoxicity in neonates caused by glutamate treatment induces neurological deficits in adults. The 14–3-3 family proteins are conserved proteins that are expressed ubiquitously in a variety of tissues. These proteins contribute to cellular processes, including signal transduction, protein synthesis, and cell cycle control. We proposed that glutamate induces neuronal cell damage by regulating 14–3-3 protein expression in newborn animals. In this study, we investigated the histopathological changes and 14–3-3 proteins expressions as a result of glutamate exposure in the neonatal cerebral cortex. Rat pups at post-natal day 7 were intraperitoneally administrated with vehicle or glutamate (10 mg/kg). Animals were sacrificed 4 h after treatment, and brain tissues were fixed for histological study. Cerebral cortices were isolated and frozen for proteomic study. We observed serious histopathological damages including shrunken dendrites and atypical neurons in glutamate-treated cerebral cortices. In addition, we identified that 14–3-3 family proteins decreased in glutamate-exposed cerebral cortices using a proteomic approach. Moreover, Western blot analysis provided results that glutamate treatment in neonates decreased 14–3-3 family proteins expressions, including the β/α, ζ/δ, γ, ε, τ, and η isoforms. 14–3-3 proteins are involved in signal transduction, metabolism, and anti-apoptotic functions. Thus, our findings suggest that glutamate induces neonatal neuronal cell damage by modulating 14–3-3 protein expression.

## Introduction

Glutamate is an excitatory neurotransmitter in the central nervous system [1]. It contributes to various physiological functions including memory and learning, synaptic transmission, and plasticity [2, 3]. A suitable glutamate concentration is very important for normal neuronal function. However, excessive glutamate induces neuronal over-excitation and neuronal excitotoxicity, and leads to neuronal cell death and neurodegeneration [4]. Glutamate overexposure increases intracellular calcium concentrations through calcium influx into the cytoplasm and activates calcium dependent intracellular enzymes. An increase in intracellular calcium leads to neuronal mitochondrial dysfunction and results in cell death [5, 6].

14–3-3 family proteins play a critical role in signal transduction, cell cycle checkpoint control, and the apoptotic pathway [7, 8]. 14–3-3 proteins are conserved scaffold proteins that are ubiquitously expressed in mammals and consist of seven isoforms, including β, γ, η, ζ, τ, ε, and σ [9, 10]. The α and δ isoforms are known as phosphorylated forms β and ζ, respectively [11]. 14–3-3 proteins are abundant in brain tissue and contribute to multiple cellular processes, such as ion channel regulation and intracellular trafficking [12]. 14–3-3 proteins interact with mitochondrial apoptotic proteins and regulate the apoptotic signal pathway. Bad is a representative pro-apoptotic protein. However, phosphorylated Bcl-2 associated death promoter (Bad) interacts with 14–3-3 and inactivates its pro-apoptotic function [13]. Our previous study demonstrated that glutamate exposure induces neonatal cerebral cortex damage by modulating a variety of proteins [14]. We propose that glutamate exposure causes neuronal cell death by regulating 14–3-3 proteins during brain development. Although the mechanism of glutamate-induced excitotoxicity has been demonstrated, little information is available regarding the changes in 14–3-3 proteins by glutamate exposure. The objective of this study is to investigate the regulation of 14–3-3 family proteins by glutamate exposure in neonatal cerebral cortex. Thus, this study investigates the changes in 14–3-3 proteins by glutamate administration in the neonatal cerebral cortex.

## Materials and methods

### Experimental animals and drug administration

Pregnant female Sprague-Dawley rats were obtained from Samtako Co. (Animal Breeding Centre, Osan, Korea) to get rat pups. Animals were kept under controlled temperature (25 °C) and lighting (12 h light / 12 h dark cycle). They were provided free access to feed and water. All animal experimental procedures were approved by the Institutional Animal Care and Use Committee of Gyeongsang National University (Approval number: GNU-190218-R0008). At post-natal 7 day, pups were divided randomly into two groups, vehicle- group and glutamate-treated group (*n* = 12 per group). Glutamate treated animals were intraperitoneally injected with glutamate (10 mg/kg, Sigma, St. Louis, MO, USA) that dissolved in normal saline. Vehicle-treated animals were injected with only normal saline as vehicle. Animals were sacrificed 4 h after administration and whole brains were removed carefully. Brains were fixed in 4% paraformaldehyde (0.1% phosphate-buffered saline, pH 7.4) for morphological studies. Brain tissues were separated from the whole brain and kept in − 70 °C for protein analysis.

### Hematoxylin and eosin staining

Fixed brain tissues were washed with running tap water for overnight. Washed tissues were dehydrated in gradient ethyl alcohol (70 to 100%) and cleaned with xylene. Tissues were embedded in paraffin with the paraffin embedding center (Leica, Westlar, Germany). Embedded tissues were cut into 4 μm thickness slices and placed over glass slides. Tissue slides were kept on slide warmer for drying, deparaffinized with xylene, and rehydrated with gradient ethyl alcohol (100 to 70%). Tissue slides were stained with Harris’ hematoxylin solution (Sigma) for 3 min and washed with running tap water for 10 min. They were dipped in 1% hydrochloric acid solution and 1% ammonia water, dipped in tap water, and stained with eosin Y solution (Sigma) for 3 min. Tissue slides were washed with tap water, dehydrated with gradient ethyl alcohol (70 to 100%), cleaned with xylene, and coverslipped with permount solution (Thermo Fisher Scientific, Waltham, MA, USA). They were observed under optical microscope (Olympus, Tokyo, Japan) and images were taken from cerebral cortex area.

### 2-dimensional gel electrophoresis

Cerebral cortices from each neonatal pups were separately lysed in lysis buffer [8 M urea, 4% 3-[(3-cholamidopropyl)dimethylammonio]-1-propanesulfonate (CHAPS), 0.2% ampholyte, 40 mM Tris-HCl]. The homogenate was centrifuged at 20,000 g for 20 min at 4 °C, and the supernatant was separated. Proteins were treated with 10% trichloroacetic acid for 30 min and centrifuged at 20,000 g for 20 min at 4 °C and protein pellets were obtained. Collected protein pellets were washed with 1 M Tris-HCl (pH 7.6) and kept at room temperature for drying. Protein pellets were mixed in sample buffer [8 M urea, 4% CHAPS, 0.2% ampholyte, 40 mM Tris-HCl, 2 μg/ml dithiothreitol (DTT)]. Protein mixtures were sonicated for 3 min, incubated for 1 h at room temperature. Mixtures were centrifuged at 15,000 g for 30 min at 4 °C and supernatants were collected. Protein concentration was determined by Bradford assay kit (Bio-Rad, Hercules, CA, USA) according to the provided instruction. First-dimensional isoelectric focusing was conducted with immobilized pH gradient (IPG) gel strips (17 cm, pH 4–7 and pH 6–9; Bio-Rad). IPG gel strips were first rehydrated in rehydrating solution [8 M urea, 2% CHAPS, 20 mM DTT, 0.5% IPG buffer, bromophenol blue] for overnight at room temperature. Strips were loaded with 50 μg protein sample and isoelectric focusing was performed through the Ettan IPGphor 3 system (GE Healthcare, Uppsala, Sweden) at 250 V for 15 min, 10,000 V for 3 h, and 10,000 to 50,000 V. IPG strips were kept in equilibration buffer [6 M urea, 30% glycerol, 2% sodium dodecyl sulfate (SDS), 50 mM Tris-HCl, bromophenol blue] with 1% DTT for 10 min and then incubated in same equilibration buffer comprising of 2.5% iodoacetamide for 10 min. Second dimensional electrophoresis was performed by loading the IPG gel strips into 7.5–17.5% gradient gel. Electrophoresis was conducted at 10 °C for overnight with 10 mA until the bromophenol blue dye went down to the bottom by Protein-II XI electrophoresis equipment (Bio-Rad). After electrophoresis, gels were fixed for 2 h in a fixing solution (12% acetic acid in 50% methanol), washed with 50% ethyl alcohol for 20 min, sensitized with 0.02% sodium thiosulfate solution for 1 min, and washed with distilled water 3 times for 1 min. Gels were stained with silver staining solution (0.2% silver nitrate, 0.03% formaldehyde) for 20 min, washed again with distilled water 3 times for 1 min, and immersed in developing solution until all the protein spots on gels were clearly visible. Silver staining reaction was finished by stop solution (1% acetic acid). Gels were scanned with Agfar ARCUS 1200™ (Agfar-Gevaert, Mortsel, Belgium), and stained protein spots were evaluated through PDQuest 2-DE analysis software (Bio-Rad). Proteins having different expressions were noted among the vehicle- and glutamate-treated groups and specific protein spots were cut off from the gel. Protein spots were destained with destaining solution (30 mM potassium hexacyanoferrate, 100 mM sodium thiosulfate) and then washed with washing solution (10% acetic acid in 50% methanol) for removal of silver stain. Spots were treated with 50 mM ammonium bicarbonate and acetonitrile for dehydration, vacuum dried with centrifuge (Biotron, Seoul, Korea) for 20 min. Dried gel spots were treated with reduction solution (10 mM DTT in 0.1 M ammonium bicarbonate) at 56 °C for 45 min, dehydrated with 0.1 M ammonium bicarbonate and acetonitrile, and once again dried in a vacuum centrifuge for 20 min. Dried proteins were digested in digestion solution (12.5 ng/ml trypsin, 0.1% octyl beta-D glycopyranside in 50 mM ammonium bicarbonate) for overnight at 37 °C. Dried spots were treated with extraction buffer (1% trifluoroacetic acid in 66% acetonitrile) for extraction of digested proteins and dried in a vacuum centrifuge for 2 h. Dried protein spots were mixed in extraction buffer and matrix solution (alpha-cyano-4-hyroxycinnamic acid and nitrocellulose in acetone) and loaded into a matrix-assisted laser desorption ionization-time (MALDI-TOF) plate (Applied Biosystem, Foster City, CA, USA). MALDI-TOF was completed with Voyager-DE STR (Applied Biosystem). Analysis of the peak results was done with NCBI and MS-FIT protein sequence database.

### Western blot analysis

Cerebral cortices were homogenized in lysis buffer (1 M Tris-HCI, 5 M sodium chloride, 0.5% sodium deoxycholate, 10% SDS, 1% sodium azide, 10% NP-40) containing leupeptin (10 μM) and phenylmethylsulfonyl fluoride (200 μM). Homogenized mixture was sonicated for 3 min and centrifuged at 15,000 g for 20 min at 4 °C. Supernatant from each sample was carefully collected and protein concentrations were measured with bicinchoninic acid (BCA) kit (Pierce, Rockford, IL, USA) according to the provided protocol. Protein 30 μg from each sample was electrophoresed in 10% SDS - polyacrylamide gels in 10 mA for 10 min, and 20 mA. These proteins were transferred into a poly-vinylidene fluoride (PVDF) membrane (Millipore, Billerica, MA, USA). Membranes were incubated in 5% skim milk (BD life science, Franklin Lakes, NJ, USA) for 1 h to block non-specific binding and washed in Tris-buffered saline comprising 0.1% Tween-20 (TBST) three times for 10 min. They were incubated for overnight with following primary antibodies: anti-14-3-3 β/α, anti-14-3-3 ζ/δ, anti-14-3-3 γ, anti-14-3-3 ε, anti-14-3-3 τ, anti-14-3-3 η (diluted 1:1000, Cell Signaling Technology, Beverly, MA, USA), and anti-β-actin (diluted 1:1000, Santa Cruz Biotechnology, Santa Cruz, CA, USA). Membranes were washed with TBST three times for 10 min and reacted with secondary antibody (1:5000, anti-mouse IgG or anti-rabbit IgG Cell Signaling Technology). Western blot analysis system (Amersham Pharmacia Biotech, Piscataway, NJ, USA) was used for visualization of protein bands on X-ray film as according to the provided protocol.

### Data analysis

Data shown are given as means ± standard error of mean (S.E.M.). Statistical analysis was completed with SigmaGel 1.0 (Jandel Scientific, San Rafael, CA, USA) and SigmaPlot 4.0 (SPSS Inc., Point Richmond, CA, USA). Statistical differences among groups were compared with one-way analysis of variance (ANOVA) followed by Student’s *t-*test. *P* < 0.05 was considered statistically significant.

## Results

**Fig. 1:**
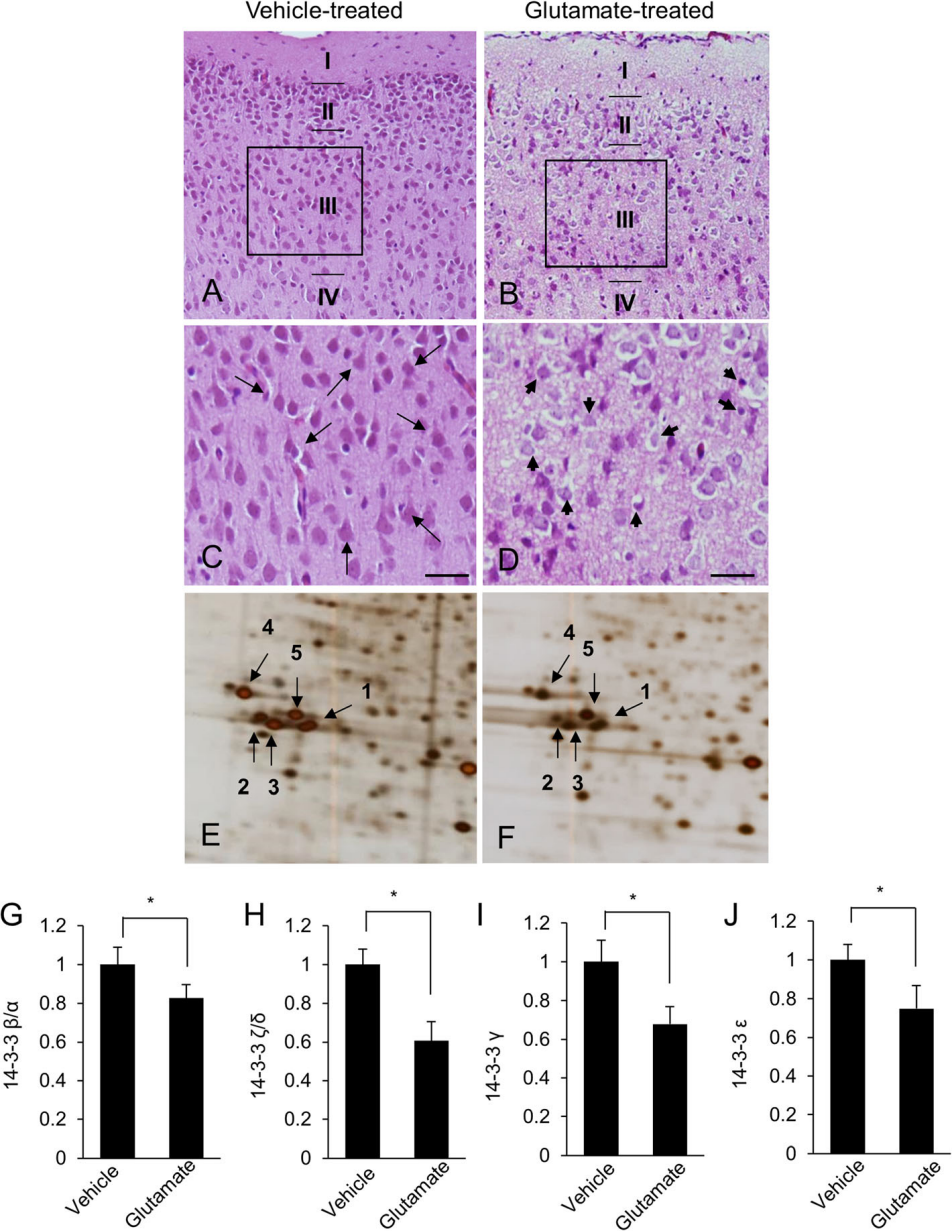
Hematoxylin and eosin stained photomicrographs (**a**-**d**), 14–3-3 β/α, 14–3-3 ζ/δ, 14–3-3 γ, and 14–3-3 ε protein spots (**e** and **f**), and graph of each spots intensity (**g**-**j**) from neonatal cerebral cortices, of vehicle- (**a**, **c** and **e**) and glutamate treated (**b**, **d** and **f**) neonatal rats. Among 6 layers of cerebral cortex, external pyramidal layer (III) was observed (**a** and **b**). Arrows indicate normal neurons with well-developed dendrites and typical pyramidal shape (**c**). Arrowheads indicate damaged neurons with shrunken dendrites and atypical round shape (**d**). Scale bar: 50 μm. Arrows and numbers indicate 14–3-3 proteins spots (**e** and **f**). (1) 14–3-3 β/α, (2, 3) 14–3-3 ζ/δ, (4) 14–3-3 γ, (5) 14–3-3 ε. Spot intensities were measured using PDQuest software. The spot intensities are reported as a ratio relative to control animals. Data (*n* = 4) are shown as mean ± S.E.M. * *P* < 0.05

**Fig. 2:**
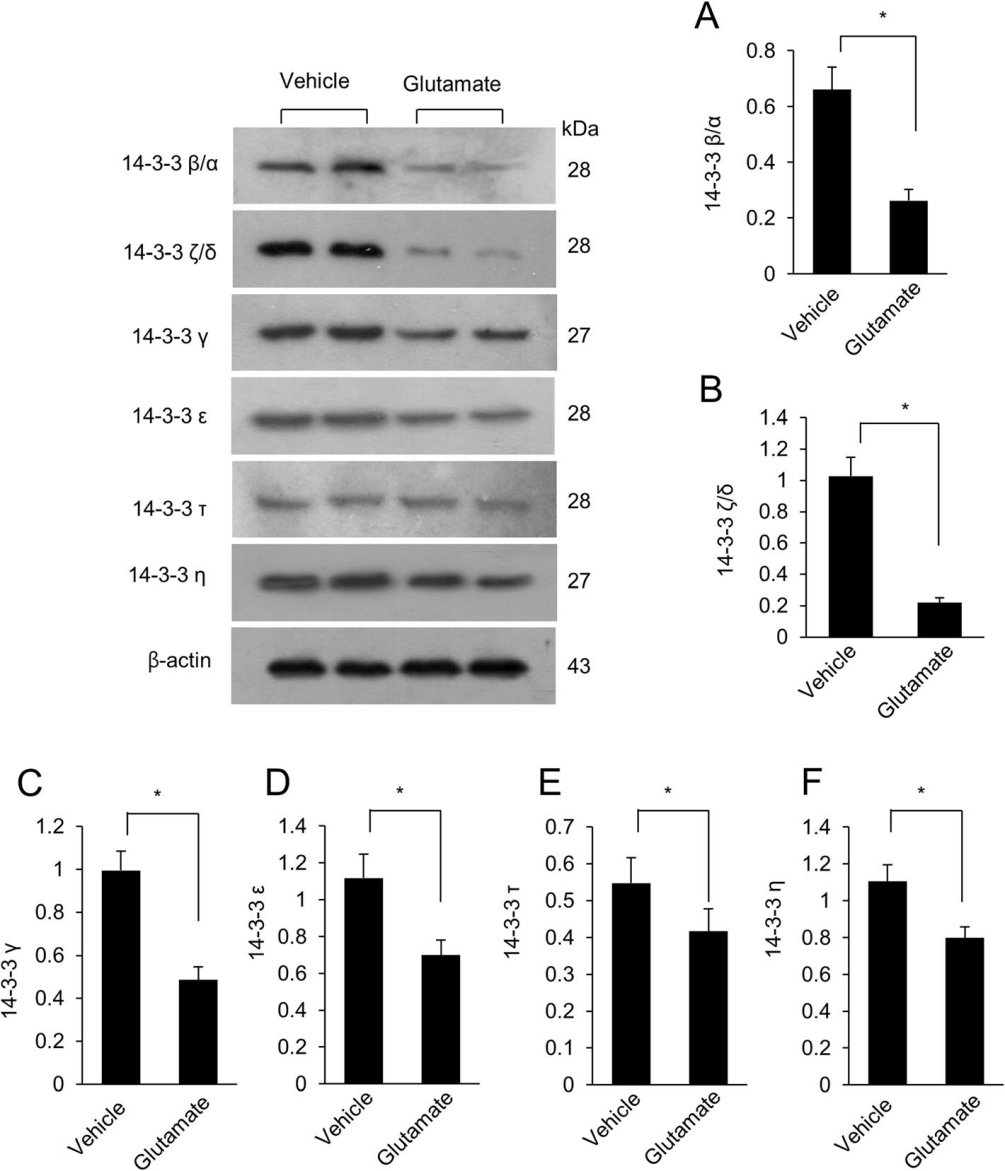
Western blot analysis of 14–3-3 β/α, 14–3-3 ζ/δ, 14–3-3 γ, 14–3-3 ε, 14–3-3 τ, and 14–3-3 η in neonatal cerebral cortices of vehicle- and glutamate-treated animals (A-F). Each lane represents an individual animal. Densitometric analysis is represented as a ratio of 14–3-3 proteins intensity to β-actin intensity. Data (*n* = 4) are shown as mean ± S.E.M. * *P* < 0.05

Glutamate treatment induces histopathological changes in the external pyramidal layer of cerebral cortex (Fig. 1). In vehicle-treated animals, we observed normal neurons of the typical pyramidal shape. These neurons had well-developed dendrites and a cell body with a large and round nucleus (Fig. 1c). However, in glutamate treated animals, we observed atypical neurons with a round shaped cell bodies and shrunken dendrites (Fig. 1d). We detected significantly changed protein spots intensity between vehicle- and glutamate-treated animals. Our results evaluated changes of the 14–3-3 β/α, 14–3-3 ζ/δ, 14–3-3 γ, and 14–3-3 ε in glutamate-treated animals. All 14–3-3 subunit protein expression levels were decreased by glutamate treatment. The expression value of the vehicle-treated group was set to 1. In the glutamate-treated group, the relative expression level of 14–3-3 β/α was 0.83 ± 0.07, 14–3-3 ζ/δ level was 0.61 ± 0.07, 14–3-3 γ level was 0.68 ± 0.07, and 14–3-3 ε level was 0.74 ± 0.07. (Fig. 1g-j). We also confirmed the decrease in these protein levels using western blot analysis (Fig. 2). 14–3-3 proteins expression levels are explained as the ratio of intensity of actin. Relative 14–3-3 β/α levels were 0.66 ± 0.08 and 0.26 ± 0.04 in vehicle- and glutamate-treated animals, respectively. Relative 14–3-3 ζ/δ levels were 1.02 ± 0.12 in vehicle-treated animals and 0.22 ± 0.03 in glutamate-treated animals, respectively, and those of 14–3-3 γ were 0.99 ± 0.09 and 0.49 ± 0.06 in vehicle- and glutamate-treated animals, respectively. Relative 14–3-3 ε levels were 1.11 ± 0.13 in vehicle-treated animals and 0.70 ± 0.08 in glutamate-treated animals, respectively. Additionally, we investigated the expression of 14–3-3 τ and 14–3-3 η. Relative 14–3-3 τ levels were 0.54 ± 0.07 and 0.41 ± 0.06 in vehicle- and glutamate-treated animals, respectively, and those of 14–3-3 η were 1.10 ± 0.09 in vehicle-treated animals and 0.79 ± 0.06 in glutamate-treated animals.

## Discussion

Glutamate plays an essential role in memory, synaptic plasticity, and neuronal development. However, glutamate overload induces excitotoxicity and causes neuronal cell damage. Glutamate excitotoxicity has been implicated in neurodegenerative disorders including Alzheimer’s disease, Parkinson’s disease, and stroke. Excessive glutamate leads to hyperexcitation of neurons and overproduction of reactive oxygen species (ROS) [15]. Increased ROS levels lead to pathological events and neuronal dysfunction. Moreover, glutamate increases intracellular calcium levels and induces mitochondrial dysfunction [16]. We previously showed that glutamate exposure induces neuronal cell damage in neonates by up-and down-regulation of specific proteins (Kang et al., 2019). Damaged neurons with atypical round shapes and shrunken dendrites were found in cells from the glutamate-treated cerebral cortex [14]. The number of anomalous neurons was increased by glutamate exposure. Our results clearly confirmed that glutamate exposure induces histopathological changes in the cerebral cortex of newborn animals. Glutamate excitotoxicity induces serious neuronal cell damage during neonatal brain development. Moreover, glutamate exposure induces unavoidable damage in the brain tissues of neonates and affects neuronal cell damage in adults [17, 18]. These results indicate a serious risk of glutamate excitotoxicity in the newborn.

We identified decreases in 14–3-3 family proteins by glutamate exposure in the neonatal cerebral cortex. 14–3-3 proteins are involved in multiple cellular processes including signal transduction, apoptosis, cell survival, and cell cycle control. 14–3-3 induces anti-apoptotic effects by interacting with pro-apoptotic proteins such as Bad, Bax, and apoptosis signal-regulating kinase 1 (ASK1) [19]. ASK1 is accepted as a pivotal component of an apoptotic signaling pathway induced by cell death stimuli such as tumor necrosis factor α and Fas; overexpression of 14–3-3 blocks ASK-1-induced apoptotic cell death [20]. The pro-apoptotic activity of ASK1 is inhibited by interaction with 14–3-3 proteins. Moreover, 14–3-3 acts as a survival factor in oxygen-glucose deprivation-induced cell death [21]. They also demonstrated that knockout of 14–3-3 increases Bax expression, whereas overexpression of 14–3-3 decreases Bax expression [21]. Finally, 14–3-3 prevents *β*-catenin/Bax-enhanced cell death in cerebral cortical neurons during ischemia [21]. Thus, 14–3-3 proteins are accepted as factors for the determination of cell fate. In this study, we showed a significant decrease in the expression of 14–3-3 proteins in the neonatal cerebral cortex following glutamate exposure. Moreover, Western blot analysis clearly confirmed the reduction of 14–3-3 protein levels. Down-regulation of 14–3-3 proteins prevents interactions between 14 and 3-3 and its binding partners, which leads to activation of their apoptotic action [22]. However, it is considered that change of 14–3-3 binding proteins is a critical event for apoptotic cell death. We did not elucidate the change of 14–3-3 binding proteins by glutamate exposure. Thus, further studies are needed to clearly show the mechanism of glutamate-induced neuronal cell damage. Although further data are need on the interactions between 14 and 3-3 and its binding proteins, our findings clearly demonstrate that glutamate exposure decreases 14–3-3 family protein levels and leads to neuronal cell damage in neonatal rats. Thus, we suggest that glutamate exposure leads to neuronal cell damage in the neonatal cerebral cortex by modulating 14–3-3 proteins.

## Conclusions

This study demonstrate that glutamate reduces 14–3-3 protein levels and induces neuronal cell damage in the cerebral cortex of newborn rats. Thus, we suggest that glutamate exposure acts as a neuropathological factor during brain development in newborns.

## Data Availability

The data that support the findings of this study are available on request from the corresponding author on reasonable request.

## Abbreviations

ASK1: Apoptosis signal-regulating kinase 1
Bad: Bcl-2 associated death promoter
BCA: Bicinchoninic acid
CHAPS: 3-[(3-cholamidopropyl)dimethylammonio]-1-propanesulfonate
DTT: Dithiothreitol
IPG: Immobilized pH gradient
MALDI-TOF: Matrix-assisted laser desorption ionization-time
NRF: National research foundation of Korea
PVDF: Poly-vinylidene fluoride
ROS: Reactive oxygen species
TBST: Tris-buffered saline containing 0.1% Tween-20
